# Coexistence of *bla*_OXA-58_ and *tet*(X) on a Novel Plasmid in *Acinetobacter* sp. From Pig in Shanghai, China

**DOI:** 10.3389/fmicb.2020.578020

**Published:** 2020-09-18

**Authors:** Jing Wang, Yan Wang, Han Wu, Zhen-Yu Wang, Peng-Cheng Shen, Yu-Qi Tian, Fan Sun, Zhi-Ming Pan, Xinan Jiao

**Affiliations:** ^1^Key Laboratory of Prevention and Control of Biological Hazard Factors (Animal Origin) for Agrifood Safety and Quality, Ministry of Agriculture of China, Yangzhou University, Yangzhou, China; ^2^Jiangsu Key Laboratory of Zoonosis/Jiangsu Co-Innovation Center for Prevention and Control of Important Animal Infectious Diseases and Zoonoses, Yangzhou University, Yangzhou, China

**Keywords:** *Acinetobacter*, *bla*_OXA-58_, plasmid, *tet*(X), tigecycline resistance

## Abstract

The purpose of this study was to characterize the complete sequence of a novel plasmid carrying tigecycline resistance gene *tet*(X) and carbapenemase gene *bla*_OXA-58_ from a swine *Acinetobacter* sp. strain SH19PTT10. Minimal inhibitory concentration (MIC) was performed using microbroth dilution method. The isolate SH19PTT10 was highly resistant (16 mg/L) to tigecycline, and also exhibited resistance to ampicillin, streptomycin, tetracycline, chloramphenicol, florfenicol, ciprofloxacin, and sulfamethoxazole/trimethoprim. Although SH19PTT10 harbored *bla*_OXA-58_, it was susceptible to cefotaxime and meropenem. The genome sequence of SH19PTT10 was determined using PacBio single-molecule real-time sequencing. Plasmid pYUSHP10-1 had a size of 174,032 bp and showed partial homology to several plasmids found in *Acinetobacter* isolates. It contained two *repA* genes, putative toxin-antitoxin systems (HipA/HipB, RelE/RelB, and BrnT/BrnA), partitioning genes (*parA* and *parB*), and heavy metal resistance-associated genes (*copA*/*copB*, *nrp*, and *czcA*/*czcD*) but the transfer region or proteins was not found. pYUSHP10-1 carried 16 resistance genes, mainly clustered in two mosaic multiresistance regions (MRRs). The first MRR contained *sul3*, *qacI*-*aadA1*-*clmA1*-*aadA2*-*bla*CARB-2-*dfrA16* cassette, *aac(3)*-*IId*, and *bla*_OXA-58_. The *bla*_OXA-58_ gene was associated with IS*Aba3*, as previously described. The second MRR is the *tet*(X) region (IS*Acsp12*-*aph(3')*-*Ia*-IS*26*-Δ*xerD*-*tet*(X)-*res*-IS*CR2*-*sul2*) related to the corresponding region in other *tet*(X)-bearing plasmids. The p*dif* sites, as well as mobile elements, play an important role in mobilization of DNA modules and plasmid evolution. Coexistence of numerous resistance genes on a single plasmid may contribute to the dissemination of these genes under pressure posed by different agents, which may explain the presence of clinically crucial resistance genes *tet*(X) and *bla*_OXA-58_ in livestock. Thus, rational drug use and continued surveillance of *tet*(X) and *bla*_OXA-58_ in livestock are warranted.

## Introduction

The genus *Acinetobacter* currently includes more than 60 species with valid species names^1^, and most of them are important nosocomial pathogens. Carbapenems are clinically crucial antimicrobial agents for treating multidrug-resistant Gram-negative pathogens, including *Acinetobacter* isolates (Asif et al., 2018; Rodríguez-Baño et al., 2018). The rapid increase in the prevalence of carbapenem-resistant *Acinetobacter* is mainly attributed to the acquisition of carbapenem-hydrolyzing class D β-lactamases (e.g., OXA-23, -40, -51, -58, and -143; Evans and Ameyes, 2014). OXA-58 has been detected in *Acinetobacter* isolates from patients, animals, and the environment from distinct geographical areas, particularly in clinics (Poirel et al., 2005; Fu et al., 2014; Feng et al., 2016; Klotz et al., 2017; Narciso et al., 2017; Chen et al., 2019; Matos et al., 2019; Suzuki et al., 2019). Though OXA-58 shows weak carbapenem-hydrolyzing activity, the insertion of other insertion sequence (IS) elements into the upstream IS*Aba3* of *bla*_OXA-58_ may provide an alternative promoter that enhances its transcription and the level of carbapenem resistance (Poirel et al., 2005; Narciso et al., 2017; Chen et al., 2019; Matos et al., 2019).

Tigecycline, belonging to the novel glycylcycline class, has been a last-resort antibiotic to treat serious infections caused by extensively drug-resistant Gram-negative bacteria, including *Acinetobacter* (Noskin, 2005; Asif et al., 2018). However, novel plasmid-mediated high-level tigecycline resistance genes *tet*(X) [former name *tet*(X3)~*tet*(X5)]^2^ have been identified in *Acinetobacter* isolates from animals and humans in China in 2019 (He et al., 2019; Wang et al., 2019). The emergence and dissemination of *tet*(X) will impair the efficacy of tigecycline in clinical treatment, thus would pose a significant threat to public health. The co-location of *tet*(X) and carbapenem resistance gene *bla*_NDM-1_ was previously described in *Acinetobacter* isolates from animals (duck, goose, and cow) and the environment (soil and sewage; Cui et al., 2020; He et al., 2020). In this study, we aimed to determine and analyze the complete sequence of a single plasmid bearing *tet*(X) [formerly designated as *tet*(X3), GenBank accession no. MK134375] and *bla*_OXA-58_ obtained from a swine *Acinetobacter* sp. strain in Shanghai, China, providing insights into the genetic structures of the plasmid and these genes.

## Materials and Methods

### Bacterial Strain and *tet*(X) Detection

In September 2019, one strain SH19PTT10 was isolated from the feces sample of a pig by Tryptic Soy Agar plate containing tigecycline (2 mg/L) from a pig farm located in Shanghai, China and was identified using 16S ribosomal RNA (rRNA) gene sequencing (Kim et al., 2010). The presence of *tet*(X) [former name *tet*(X3), *tet*(X4), and *tet*(X5)] was detected by PCR and sequencing (He et al., 2019; Wang et al., 2019).

### Antimicrobial Susceptibility Testing

The isolate SH19PTT10 was tested for minimal inhibitory concentrations (MICs) of ampicillin, cefotaxime, meropenem, amikacin, streptomycin, tetracycline, minocycline, tigecycline, chloramphenicol, florfenicol, ciprofloxacin, colistin, and sulfamethoxazole/trimethoprim using microbroth dilution method recommended by the guidelines of the Clinical and Laboratory Standards Institute [CLSI, Wayne, PA, United States; Clinical and Laboratory Standards Institute (CLSI), 2012]. The results were interpreted according to CLSI M100, 28th edition [Clinical and Laboratory Standards Institute (CLSI), 2018]. Tigecycline (≧1 mg/L), streptomycin (≧32 mg/L), and florfenicol (≧32 mg/L) were interpreted according to the clinical breakpoint or epidemiological cutoff values for *Escherichia coli* set by EUCAST^3^. The *E. coli* strain ATCC 25922 was used for quality control.

### Conjugation/Transformation Experiments

Conjugation experiments were performed using streptomycin-resistant *E. coli* C600 as the recipient strain, as previously described (Chen et al., 2007). Transconjugants were selected using 2 mg/L tigecycline and 3,000 mg/L streptomycin. Transformation was carried out by heat-shock and electroporation using *E. coli* DH5α and *Acinetobacter baumannii* ATCC 19606. Transformants were selected by 2 mg/L tigecycline.

### Whole Genome Sequencing and Analysis

The whole genome of SH19PTT10 was extracted and sequenced using PacBio single-molecule real-time sequencing (RSII platform, Pacific Biosciences, Menlo Park, CA, United States). Raw sequence data were introduced into the non-hybrid hierarchical genome assembly process (HGAP version 4). The 16S rRNA gene sequences of SH19PTT10 and other representatives across the genus *Acinetobacter* were aligned using ClustalW, and a phylogenetic tree was constructed by the neighbor joining algorithm using MEGA 7.0 (Kumar et al., 2008). The plasmid sequence was analyzed and annotated using the RAST server (Aziz et al., 2008), ResFinder 3.2 (Zankari et al., 2012), ISfinder (Siguier et al., 2006), PlasmidFinder (Carattoli et al., 2014), BLAST^4^, and Gene Construction Kit 4.5 (Textco BioSoftware, Inc., Raleigh, NC, United States). The replicase genes (*rep*) of plasmids from SH19PTT10 were assigned to a group according to the typing scheme for plasmids in *A. baumannii* (Bertini et al., 2010). Plasmid pYUSHP10-1 was compared with other plasmids using BLASTn and BRIG (Alikhan et al., 2011).

### Nucleotide Sequence Accession Number

The complete sequence of pYUSHP10-1 was deposited in GenBank under the accession number MT107270.

## Results and Discussion

### *tet*(X)-Encoding *Acinetobacter* sp. Strain SH19PTT10

The strain SH19PTT10 was positive for *tet*(X) [formerly designated as *tet*(X3), MK134375] and was highly resistant (16 mg/L) to tigecycline. SH19PTT10 also showed resistance to ampicillin, streptomycin, tetracycline, chloramphenicol, florfenicol, ciprofloxacin, and sulfamethoxazole/trimethoprim but was susceptible to cefotaxime, meropenem, amikacin, minocycline, and colistin (Table 1). The 16S rRNA gene sequencing showed SH19PTT10 belonging to *Acinetobacter* but not to any described species, thus whole genome sequencing (WGS) was further performed.

**Table 1:** Results of antimicrobial susceptibility test.

Antimicrobial agents	MIC (mg/L)	Interpretation
ampicillin	>128	R
cefotaxime	4	S
meropenem	0.5	S
amikacin	1	S
streptomycin	>128	R
tetracycline	>128	R
minocycline	≤2	S
tigecycline	16	R
chloramphenicol	128	R
florfenicol	128	R
ciprofloxacin	>8	R
colistin	0.25	S
sulfamethoxazole/trimethoprim	>32	R

SH19PTT10 consisted of a 3,440,498-bp chromosome and five plasmids designated as pYUSHP10-1 to pYUSHP10-5, ranging from 5.7 to 174 kb (Supplementary Table S1). Among them, pYUSHP10-1, the largest plasmid found in SH19PTT10, carried numerous resistance genes, particularly tigecycline resistance gene *tet*(X) and carbapenemase gene *bla*_OXA-58_; pYUSHP10-3 also carried resistance gene *tet*(39) (Supplementary Table S1). The *tet*(X)-carrying plasmid pYUSHP10-1 was failed to transfer to *E. coli* C600/DH5α by conjugation/transformation or *A. baumannii* ATCC 19606 by transformation. The complete sequence of the 16S rRNA gene exhibited 98.76% identity (1515/1534) with *A. cumulans* strain WCHAc060092 (CP035934) and 98.37% identity (1509/1534) with *Acinetobacter haemolyticus* strains AN54 (CP041224). The neighbor-joining tree based on partial 16S rRNA gene sequences within *Acinetobacter* suggested that the isolate SH19PTT10 may take a distinct position within the genus; however, the branch support for SH19PTT10 was low (bootstrap value 36%; Supplementary Figure S1).

### The Backbone of *tet*(X)‐ and *bla*_OXA-58_-Bearing Plasmid pYUSHP10-1

**Figure 1:**
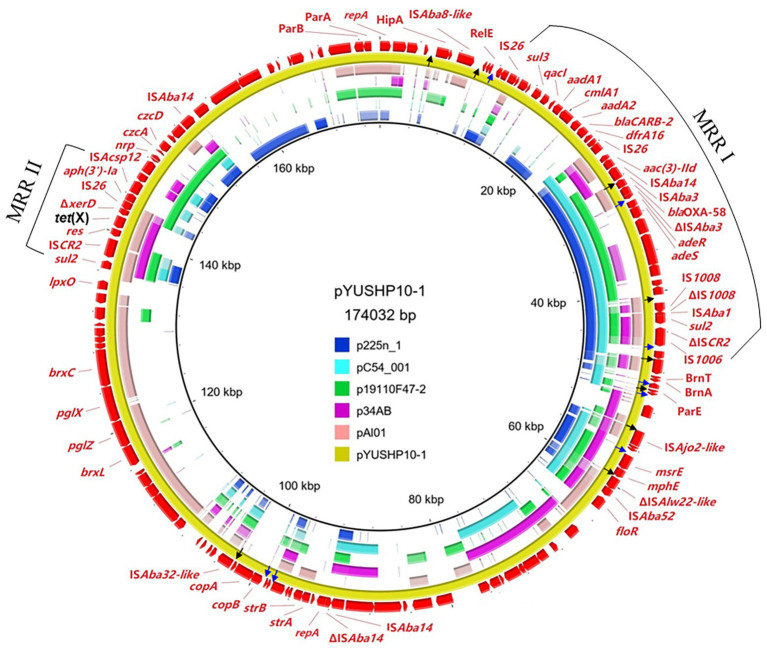
Sequence comparison of plasmids pYUSHP10-1 with plasmids p225n_1 (GenBank accession number KT852971), pC54_001 (CP042365), p19110F47-2 (CP046044), p34AB (MK134375), and pAl01 (CP044019) using BRIG. The reference sequence pYUSHP10-1 is indicated in red in the outer circle. The arrows in black color indicate p*dif* sites (XerD-XerC) and arrows in blue color indicate p*dif* sites (XerD-XerC).

The plasmid pYUSHP10-1 had a size of 174,032 bp with a 41.32% GC content; it could not be assigned to any previously known incompatibility group. pYUSHP10-1 showed partial homology to several plasmids obtained from *Acinetobacter* isolates, such as OXA-58-encoding plasmids p225n_1 (KT852971, *A. baumannii*, patient, Vietnam), pC54_001 (CP042365, *Acinetobacter pittii*, patient, Australia), and p19110F47-2 (CP046044, *Acinetobacter towneri*, pig, China), as well as *tet*(X)-carrying plasmids p34AB (MK134375, *A. baumannii*, swine, China) and pAl01 (CP044019, *Acinetobacter indicus*, feces, China; 29–41% coverage, 97.3–99.9% nucleotide identity; Figure 1).

pYUSHP10-1 contained two *repA* genes, both of them belonged to the Rep_3 type family (pfam01051). The first *repA* (positions 1–1068) shared highly similarity (>99.9%) with those of tigecycline-resistant plasmids p18TQ-X3 (CP045132, 1067/1068) and pAB17H194-1 (CP040912, 1067/1068) obtained from *Acinetobacter* strains in China. The second *repA* (positions 92451–93302) was identical to those of plasmids pAHTJR1 (CP038010, *A. haemolyticus*, patient) and pBXX1-9 (CP010351, *Acinetobacter johnsonii*, hospital sewage; Feng et al., 2016) in China. In addition, pYUSHP10-1 harbored multiple putative toxin-antitoxin systems, such as HipA/HipB, RelE/RelB, and BrnT/BrnA, and the partitioning genes (*parA* and *parB*), which may ensure plasmid maintenance at cell division (Zielenkiewicz and Ceglowski, 2001). However, the transfer region or proteins for plasmid mobilization was not found in pYUSHP10-1. Putative part of the BREX system (*brxC-pglX-pglZ-brxL*) was identified in plasmid pYUSHP10-1 (Figure 1), which has been reported as a novel phage defense system which confers resistance to a broad range of phages (Goldfarb et al., 2015). Similar structure was also found in plasmids from *Acinetobacter*, such as pOXA58_010062 (CP033131, *Acinetobacter wuhouensis*, sewage, China) with 91% coverage and 96.21% identity and pAl01 shared 93% coverage and 93.93% identity. Additionally, heavy metal resistance-associated genes were also detected in pYUSHP10-1, such as *copA*/*copB* (copper resistance), *nrp* (nickel resistance), and *czcA*/*czcD* (cobalt-zinc-cadmium resistance; Figure 1).

### The p*dif* Sites of pYUSHP10-1

Recently, numerous plasmids from various *Acinetobacter* species have been identified to contain 28-bp p*dif* site, consisting of 11-bp inversely-oriented binding sites for the XerC and XerD recombinases separated by a spacer of 6 bp (Supplementary Table S2; D’Andrea et al., 2009; Merino et al., 2010; Blackwell and Hall, 2017; Cameranesi et al., 2018). We also found 17 p*dif* sites on pYUSHP10-1 (Figure 1; Supplementary Tables S2 and S3). The modules flanked by inversely-oriented p*dif* sites either with the XerC sites internal (D/C and C/D) or the XerD sites internal (C/D and D/C) are identified as *dif* module and may be able to mobilize mediated by p*dif* sites using XerC-XerD recombination system (D’Andrea et al., 2009; Merino et al., 2010; Blackwell and Hall, 2017; Cameranesi et al., 2018). For example, one module in pYUSHP10-1 consisting of IS*Ajo2-like* and putative toxin-antitoxin system BrnT/BrnA, was surrounded by inversely-oriented p*dif* sites (XerD/C and XerC/D; Supplementary Figure S2). This *dif* module was adjacent to a hypothetical protein and a further *dif* module carrying another putative toxin-antitoxin system encoding ParE toxin and helix-turn-helix family protein (Supplementary Figure S2). Interestingly, the p*dif* sites flanking two *dif* modules were also surrounding the hypothetical protein in the opposite orientation, making it being a putative *dif* module that may be mobile (Supplementary Figure S2). Different *dif* module combinations were found in some *Acinetobacter* plasmids such as pABIR (EU294228), pJ9-3 (CP041590), and pM131-2 (JX101647), indicating that the presence of the p*dif* sites facilitated the mobilization of discrete DNA segment through multiple events (Supplementary Figure S2). Additional *dif* modules identified in pYUSHP10-1 mostly include toxin-antitoxin systems, and *copA*/*copB* were also found in *dif* module (Figure 1).

### pYUSHP10-1 Carrying Resistance Genes and *dif* Modules

**Figure 2:**
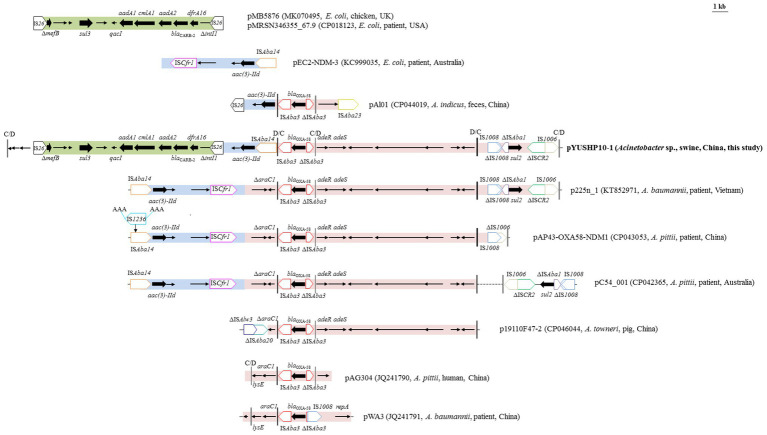
Genetic organization of the multiresistance region I of plasmid pYUSHP10-1, and structural comparison with other plasmids. The extents and directions of antibiotic resistance (thick arrows) and other genes are indicated. Δ indicates a truncated gene or mobile element. Insertion sequences (ISs) are shown as boxes labeled with their name. Labeled vertical arrows with IS box indicate the insertion site of IS element. Direct repeats are indicated by arrows and sequences. Vertical bars in black color indicate p*dif* sites (XerD-XerC) and vertical bars in gray color indicate p*dif* sites (XerD-XerC).

pYUSHP10-1 contained 16 resistance genes, which were mainly clustered in two mosaic multiresistance regions (MRRs; Figure 1). The first MRR carried an approximately 32.2-kb segment consisting of three parts. The first of these (~11.6 kb) was bounded at both ends by IS*26* and comprised a truncated *mefB* (encoding macrolide efflux protein), *sul3* (sulfonamide resistance), and an incomplete class 1 integron with Δ*intI1* and the *qacI*-*aadA1*-*clmA1*-*aadA2*-*bla*_CARB-2_-*dfrA16* cassette array (Figure 2). This fragment showed 99.9% identity to those of plasmids from *E. coli* such as pMB5876 (MK070495) and pMRSN346355_67.9 (CP018123), suggesting that pYUSHP10-1 may capture this segment from *E. coli* plasmids by IS*26*-mediated transposition or homologous recombination (Figure 2).

The second part (3,296 bp) contained an open reading frame (ORF) encoding AAA family ATPase, aminoglycoside resistance gene *aac(3)*-*IId*, and mobile element IS*Aba14* (Figure 2). It was highly similar (>99%) to the corresponding regions of multiple plasmids found in *Acinetobacter* and *Enterobacteriaceae* isolates, such as *Acinetobacter* plasmids p225n_1 and pC54_001 and *E. coli* plasmid pEC2-NDM-3 (KC999035). Interestingly, the p*dif* site adjacent to IS*Aba14* and the p*dif* site upstream of IS*26* were in inverse orientation, suggesting that the acquisition of ~16.5 kb segment including two hypothetical proteins and the first and second parts of MRR I in pYUSHP10-1 was possibly mediated by the p*dif* sites *via* site-specific recombination (Figure 2).

The last part of MRR I (17,301 bp) was highly similar to those of *Acinetobacter* plasmids p225n_1 (one single nucleotide polymorphism), p19110F47-2, pC54_001, and pAP43-OXA58-NDM1 with additional deletions (Figure 2). The primary component of this fragment is the *bla*_OXA-58_ region. As previously described (Fu et al., 2014; Feng et al., 2016; Klotz et al., 2017), *bla*_OXA-58_ was flanked by two copies of IS*Aba3* with opposite orientation, although the upstream IS*Aba3* was incomplete in pYUSHP10-1 (Figure 2). OXA-58 shows weak activity against the carbapenems and is unable to hydrolyze some cephalosporin such as ceftazidime and cefotaxime (Poirel et al., 2005). The insertion of other IS elements, such as IS*Aba825*, IS*Our1*, IS*1008*, and IS*1006* into the upstream IS*Aba3* may provide a promoter to enhance the expression of OXA-58 and leading to the carbapenem resistance (Fu et al., 2014; Narciso et al., 2017; Chen et al., 2019; Matos et al., 2019). In this study, we did not observe insertion of IS element into IS*Aba3*, suggesting the lacking of a putative promoter for *bla*_OXA-58_ overexpression, thus the strain SH19PTT10 showed susceptibility to cefotaxime (4 mg/L) and meropenem (0.5 mg/L).

The *bla*_OXA-58_ region has been frequently bracketed by two Re27 sequences, recognized as p*dif* sites later (D’Andrea et al., 2009), which have been shown to mediate the mobilization of *bla*_OXA-58_ (Poirel and Nordmann, 2006). In this study, two p*dif* sites in inverse orientation (XerD-XerC and XerC-XerD) were identified flanking the 2,257-bp segment containing IS*Aba3*-*bla*_OXA-58_-ΔIS*Aba3* arrangement in pYUSHP10-1, as observed in numerous *Acinetobacter* plasmids (Figure 2). The downstream IS*Aba3* was commonly followed by the putative transcriptional regulator gene *araC1* and threonine efflux protein gene *lysE* in *Acinetobacter* isolates (Figure 2; Fu et al., 2014; Feng et al., 2016; Matos et al., 2019). An additional p*dif* site was found adjacent to *lysE* (Figure 2), which may account for the mobilization of this segment together with the *bla*_OXA-58_ *dif* module. However, the conserved structure *araC1*-*lysE* was not found in pYUSHP10-1. A truncated *araC1* was identified in p225n_1, pAP43-OXA58-NDM1, and pC54_001, possibly due to homologous recombination between *araC1* and the downstream putative gene encoding site-specific integrase, which shared a sequence of 6 bp (TAAGTT) that might be the site of recombination (Figure 2). The same Δ*araC1* was also observed in p19110F47-2, which truncated by an incomplete IS*Aba20* (Figure 2).

The genetic contexts downstream of IS*Aba3*-*bla*_OXA-58_-ΔIS*Aba3* is diverse in *Acinetobacter* (Figure 2; Fu et al., 2014; Feng et al., 2016; Klotz et al., 2017; Matos et al., 2019). In pYUSHP10-1, several putative ORFs encoding two-component regulatory system AdeR/AdeS, RND family efflux transporters, LysE family protein, and AraC family transcriptional regulator were identified (Figure 2). In addition, a ~5.1 kb segment was further present downstream consisting of sulfonamide resistance gene *sul2* and several intact or truncated insertion sequences, including IS*1008*, IS*Aba1*, IS*CR2*, and IS*1006* (Figure 2). Two p*dif* sites in inverse orientation (XerD-XerC and XerC-XerD) were found within this segment to create a *dif* module (Figure 2). The first one disclosed an additional *adeR*/*adeS dif* module with the p*dif* site downstream of ΔIS*Aba3*, which may readily explain its co-transfer with the *bla*_OXA-58_ *dif* module in several plasmids, e.g., p19119F47-2 (Figure 2). The structure IS*Aba1*-*sul2*-ΔIS*CR2*-IS*1006* was also observed in many plasmids found in *Acinetobacter* and *Enterobacteriaceae* isolates, such as pMCR_WCHEC050613 (CP019214, *E. coli*, sewage, China), pOXA58_005069 (CP026086, *A. pittii*, patient, China), and 1205p1 (CP012141, *Shigella flexneri*, China), although IS*Aba1* was truncated by IS*1008* at 3' end in pYUSHP10-1.

**Figure 3:**
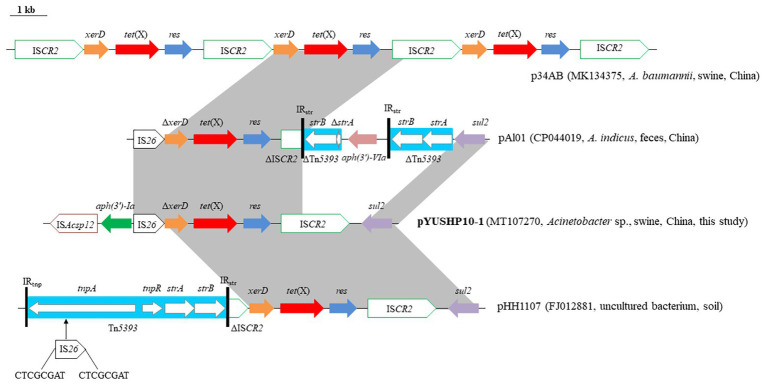
Genetic organization of the multiresistance region II of plasmid pYUSHP10-1, and structural comparison with other plasmids. Arrows indicate the position of the genes and the direction. Regions with >99% homology are shaded in gray. Δ indicates a truncated gene or mobile element. ISs are shown as boxes labeled with their name. Labeled vertical arrows with IS box indicate the insertion site of IS element. Direct repeats are indicated by arrows and sequences. Tall bars represent the 81 bp inverted repeats (IRs) of Tn*5393*.

The second MRR module (~9.2 kb) is the *tet*(X) region. In pYUSHP10-1, *tet*(X) had a single nucleotide substitution at position 21 (A21) compared with that of the first described *tet*(X)-bearing plasmid p34AB from swine *A. baumanii* (He et al., 2019) but it did not result in any amino acid change. In p34AB, *tet*(X) was located within the arrangement IS*CR2*-*xerD*-*tet*(X)-*res*-IS*CR2* with three copies (He et al., 2019). pYUSHP10-1 possessed a structure [IS*Acsp12*-*aph(3')*-*Ia*-IS*26*-Δ*xerD*-*tet*(X)-*res*-IS*CR2-sul2*] closely related to those of p34AB, pHH1107 (FJ012881, soil), and pAl01 (Figure 3). A new IS element was identified adjacent to the aminoglycoside resistance gene *aph(3')*-*Ia* and showed 87% identity to IS*Aba21*. It was 1,274 bp and had 36-bp imperfect IR, and it was designated as IS*Acsp12* in ISFinder database^5^. The fragment (*res*-IS*CR2*-*sul2*) located downstream of *tet*(X) was similar to the corresponding region in pHH1107, differed by only one nucleotide change within IS*CR2*. In pYUSHP10-1, *xerD* which encoded a recombinase was truncated by IS*26* element at the 5' end; it may explain the absence of the upstream IS*CR2* observed in p34AB. A similar segment was found in pAl01 except that *aph(3')*-*Ia* was present upstream of IS*26*. In pHH1107, the upstream IS*CR2* was truncated by a complete transposon Tn*5393* at the 5′ end, which contained streptomycin resistance genes *strA*/*strB* and was interrupted by IS*26* at *tnpA* of Tn*5393*, generated 8-bp direct repeats (CTCGCGAT; Figure 3).

The horizontal transfer of *tet*(X) is likely to be associated with IS*CR2* (He et al., 2019). The generation of a circular molecule [IS*CR2*-*xerD*-*tet*(X)-*res*] by recombination between the two copies of IS*CR2* in the same orientation could lead to the insertion of the *tet*(X) module (He et al., 2019). Although two copies of IS*CR2* were only intact in p34AB, the formation of different but related *tet*(X) structures may have resulted from additional molecular events mediated by mobile elements (e.g., IS*26* and Tn*5393*) *via* transposition or homologous recombination and have the potential to evolve diverse *tet*(X) genetic contexts.

pYUSHP10-1 also carried other resistance genes, including macrolide resistance genes *mph*(E)/*msr*(E), florfenicol resistance gene *floR*, and streptomycin resistance genes *strA*/*strB* (Figure 1). As previously described (Blackwell and Hall, 2017), a 2,950-bp segment including the macrolide resistance genes *mph*(E)/*msr*(E) was surrounded by two p*dif* sites in inverse orientation (Figure 1), further suggesting that the p*dif* sites may mediate *mph*(E)/*msr*(E) mobilization in *Acinetobacter* plasmids. Furthermore, many intact or truncated insertion sequences were present in the backbone of pYUSHP10-1, such as IS*Aba14* (Figure 1). These IS elements may enable this plasmid to capture more genes and evolve through IS-mediated recombination events.

## Conclusion

We report the isolation and genetic characterization of an *Acinetobacter* sp. strain exhibited multiresistance phenotype, due to the acquisition of a novel plasmid carrying 16 resistance genes, including tigecycline resistance gene *tet*(X) and carbepenemase gene *bla*_OXA-58_. The *Acinetobacter* sp. may serve as an important reservoir of antimicrobial resistance genes. Coexistence of numerous resistance genes on a single plasmid may facilitate its dissemination and persistence under different selection pressure, which may explain the presence of clinically crucial antibiotic resistance genes *tet*(X) and *bla*_OXA-58_ in livestock. Additionally, at least two different mechanisms, site-specific recombination *via* the p*dif* sites and transposition of mobile elements (e.g., IS*CR2* and IS*26*) could account for the acquisition of DNA modules containing resistance structures and/or other genes in pYUSHP10-1. It highlights the ability of resistance structures to be captured by multiple events and their capacity to evolve during horizontal transfer.

## Data Availability Statement

The datasets presented in this study can be found in online repositories. The names of the repository/repositories and accession number(s) can be found in the article/Supplementary Material.

## Author Contributions

Z-MP, XJ, and JW conceived the study. YW, HW, P-CS, JW, Y-QT, and FS carried out the experiments. JW and Z-YW analyzed the data. JW wrote the manuscript. Z-MP and XJ revised the manuscript. All authors contributed to the article and approved the submitted version.

### Conflict of Interest

The authors declare that the research was conducted in the absence of any commercial or financial relationships that could be construed as a potential conflict of interest.

## Footnotes

^1^https://apps.szu.cz/anemec/Classification.pdf

^2^According to the standards of the nomenclature center (http://faculty.washington.edu/marilynr/), all *tet*(X) genes [former name *tet*(X1)-*tet*(X5)] at present can only be designated as *tet*(X).

^3^https://www.eucast.org

^4^http://blast.ncbi.nlm.nih.gov/Blast.cgi

^5^https://www-is.biotoul.fr
