# Supplementary material for: Coexistence of blaOXA-58 and tet(X) on a Novel Plasmid in Acinetobacter sp. From Pig in Shanghai, China
Source: Front Microbiol. 2020 Sep 18;11:578020. doi: 10.3389/fmicb.2020.578020 (PMC7530245; doi:10.3389/fmicb.2020.578020)
Supplement: Supplementary file 1 [file Data_Sheet_1.pdf]

Table S1. Plasmids of SH19PTT10

Plasmid	Size (bp)	Resistance genes	Closest match of *rep*: plasmid (identity)	*rep* group
pYUSHP10-1	174,032	*aac(3)-IId*, *aadA1*, *aadA2*, *aph(3')-Ia*, *bla*$_{CARB-2}$, *bla*$_{OXA-58}$, *cmlA1*, *dfrA16*, *floR*, *mph*(E), *msr*(E), *strA*, *strB*, *sul2*, *sul3*, *tet*(X)	first *rep*: pAB17H194-1 (99.9%)<br>second *rep*: pXBB1-9 (100.0%)	new<br>new
pYUSHP10-2	17,299	none	pALWED1.3 (99.6%)	new
pYUSHP10-3	8,312	*tet*(39)	p537 (95.9%)	GR5
pYUSHP10-4	8,168	none	/	new
pYUSHP10-5	5,738	none	*rep* undetermined	/

Table S2 Number of p*dif* sites identified in *Acinetobacter* plasmids

**Plasmid[a]**	**Size (bp)**	**p*dif* sites**	**Source**	**Accession number**
pS30-1	18,234	8	*A. baumannii*	KY617771
pXBB1-9	398,857	12	*A. johnsonii*	CP010351
pABIR	29,823	13	*A. baumannii*	EU294228
p255n_1	92,939	9	*A. baumannii*	KT852971
pC54_001	256,887	9	*A. pittii*	CP042365
pAP43-OXA58-NDM1	268,263	10	*A. pittii*	CP043053
p19110F47-2	143,035	11	*A. towneri*	CP046044
p34AB	277,864	6	*A. baumannii*	MK134375
pAI01	116,992	4	*A. indicus*	CP044019
**pYUSHP10-1**	174,032	17	*Acinetobacter* sp.	MT107270

a. Plasmid with name in bold typeface is obtained in this study.

Table S3 The p*dif* sites found in pYUSHP10-1

p*dif* sites[a]	Site sequence (5'-3')[b] XerC-XerD	Position in pYUSHP10-1 (bp)
p*dif*-1*	ACTTCGTATAATAGTGATTATGTTAAAT	4,962-4,989
p*dif*-2*	ATTGCGTATAACCACCATTATGTTAAAT	9,940-9,967
p*dif*-3	AATTCGTATAACCACCATTATGTTAAAT	11,600-11,627
p*dif*-4*	ATTTTGTATAAGGTGTATTATGTTAATT	28,179-28,206
p*dif*-5	GTTTCGTATAACAGCCATTATGTTAAAT	30,464-30,491
p*dif*-6*	ATTACGTATAACGTGTATTATGTTAATT	40,394-40,421
p*dif*-7	ATTACGTATAAGGTGTATTATGTTAATT	45,473-45,500
p*dif*-8*	ATTTCGTATAACGCCCATTATGTTAAAT	46,710-46,737
p*dif*-9	ACTTCGCATAACGCCCATTATGTTAATT	49,053-49,080
p*dif*-10*	ATTTCGTATAACCGCCATTATGTTAAGT	49,695-49,722
p*dif*-11	ATTTCGTATAAGGTGTATTATGTTAATT	50,419-50,446
p*dif*-12*	GTTTCGCATAACAGCCATTATGTTAAAT	54,060-54,087
p*dif*-13	ATTTCGTATAAGGTGTATTATGTTAATT	56,277-56,304
p*dif*-14*	ACTTCACATAAGAAATTTTATGTTAAAT	59,255-59,282
p*dif*-15	ATTTCGCATAAGAGATTTTATGTTAAAT	98,080- 98,107
p*dif*-16	GTTTCGTATAAGCTCTATTATGTTAAAT	98,970-98,997
p*dif*-17*	ATTTCGTATAAGGTGTATTATGTTAATT	102,476-102,503

a. The p*dif* site (XerD-XerC) is listed as reverse complementary sequence and was indicated with asterisk.

b. The XerC binding site is shown in blue color, and the XerD binding site is shown in red color. The consensus sequence is underlined.

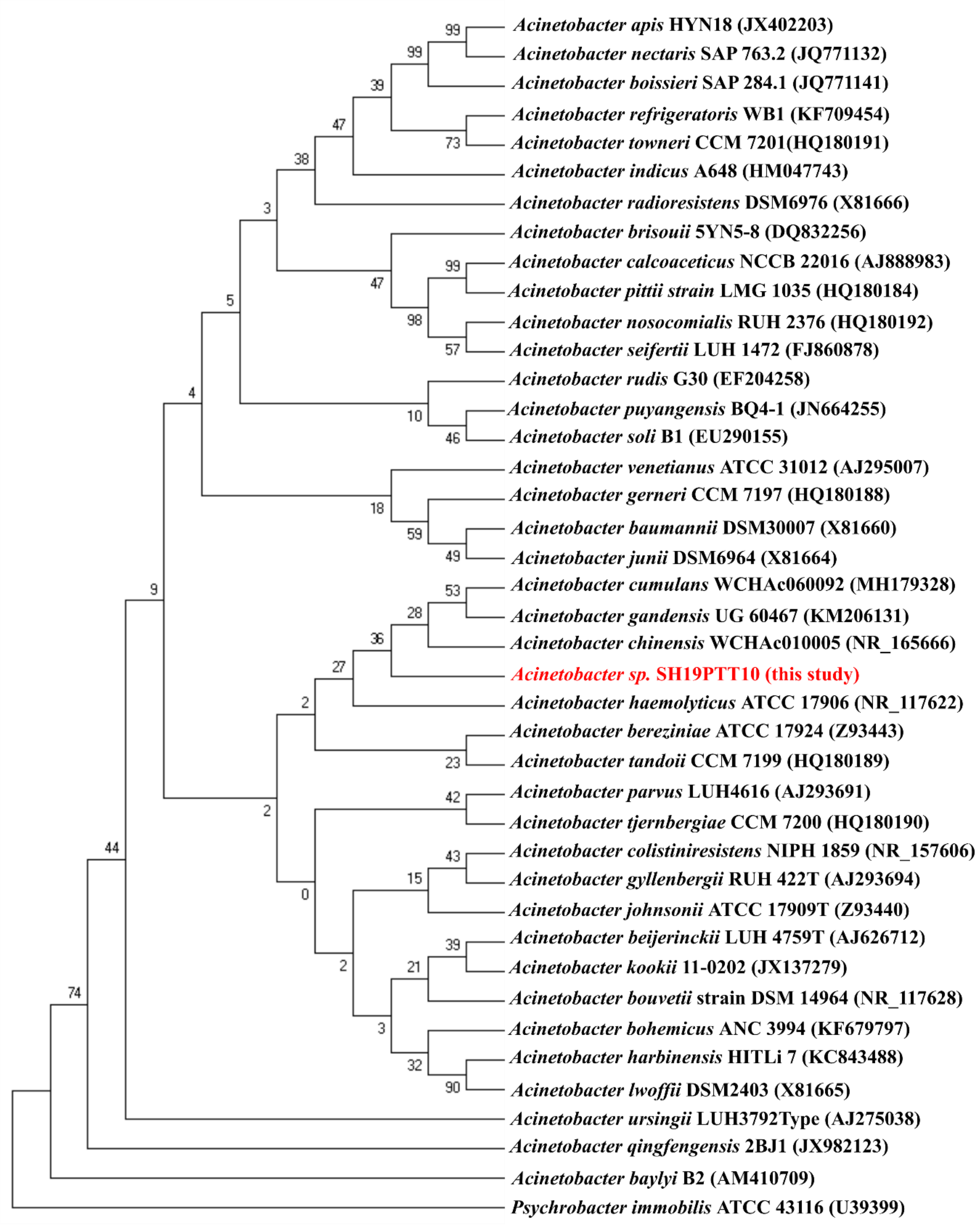

Figure S1 Neighbour-joining phylogenetic reconstruction from 16S rRNA gene sequences of SH19PTT10 and other representatives across the genus *Acinetobacter*. The percentage of replicate trees in which the associated taxa clustered together in the bootstrap test (1000 replicates) is shown next to the branches. *Psychrobacter immobilis* ATCC 43116 (GenBank accession no. U39399) was used as an outgroup.

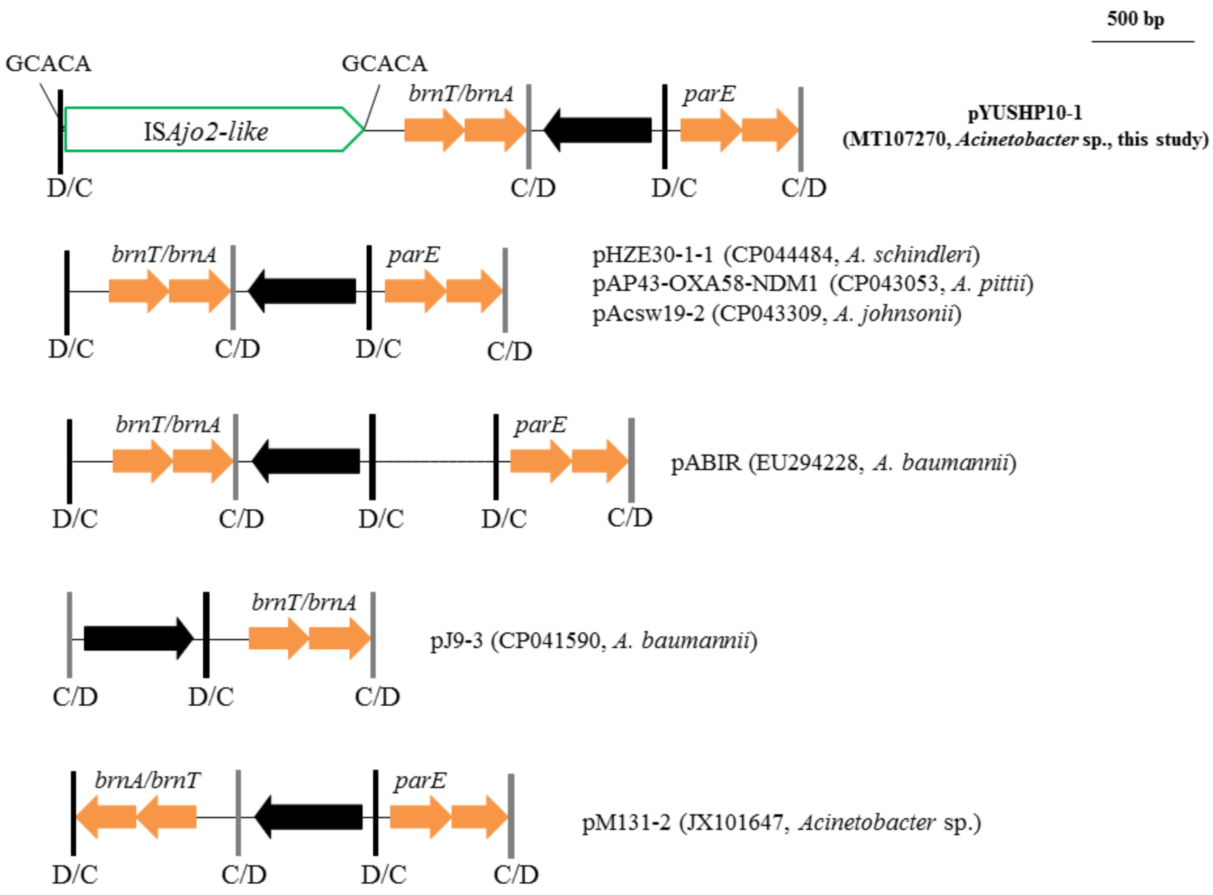

Figure S2 Comparison of sequences containing *brnT*/*brnA dif* module. Arrows indicate the extent and direction of genes. The hypothetical protein is shown in black color. The toxin-antitoxin system is shown in orange color. IS element is shown as box labeled with its name. Direct repeats are indicated by arrows and sequences. Vertical bars in black color indicate p*dif* sites (XerD-XerC) and vertical bars in grey color indicate p*dif* sites (XerD-XerC).
